# Bloodstream Infection by *Saccharomyces cerevisiae* in Two COVID-19 Patients after Receiving Supplementation of *Saccharomyces* in the ICU

**DOI:** 10.3390/jof6030098

**Published:** 2020-06-30

**Authors:** Ioannis Ventoulis, Theopisti Sarmourli, Pinelopi Amoiridou, Paraskevi Mantzana, Maria Exindari, Georgia Gioula, Timoleon-Achilleas Vyzantiadis

**Affiliations:** 1 Intensive Care Unit, “Bodossakio” General Hospital of Ptolemaida, 50200 Ptolemaida, Greece; ventoulis@hotmail.com (I.V.); pamoirid@yahoo.gr (P.A.); 2 Department of Microbiology, Medical School, Aristotle University of Thessaloniki, 54124 Thessaloniki, Greece; p.sarmourli@gmail.com (T.S.); mexidari@auth.gr (M.E.); ggioula@med.auth.gr (G.G.); 3 Department of Microbiology, AHEPA Hospital, Aristotle University of Thessaloniki, 54636 Thessaloniki, Greece; vimantzana@gmail.com

**Keywords:** COVID-19, fungaemia, *Saccharomyces*, co-infections

## Abstract

Co-infections have an unknown impact on the morbidity and mortality of the new clinical syndrome called coronavirus disease 2019 (COVID-19). The syndrome is caused by the new pandemic coronavirus SARS-CoV-2 and it is probably connected with severe traces in the elements of the immune system. Apart from possible *Aspergillus* infections, particularly in patients with acute respiratory distress syndrome (ARDS), other fungal infections could occur, probably more easily, due to the immunological dysregulation and the critical condition of these patients. Probiotic preparations of *Saccharomyces* are broadly used for the prevention of antibiotic-associated complications, especially in the intensive care units (ICU). On the other hand, *Saccharomyces* organisms are reported as agents of invasive infection in immunocompromised or critically ill patients. We report two cases of bloodstream infection by *Saccharomyces* in two patients hospitalised in the ICU, due to severe COVID-19, after *Saccharomyces* supplementation.

## 1. Introduction

The new pandemic caused by the coronavirus SARS-CoV-2 has evolved as a major health threat and has been connected to a big number of deaths worldwide, while the future spread of the disease is more or less unknown.

While it is already known that patients with co-morbidities and underlying diseases present poorer clinical outcomes [1], the frequency of co-infections and their impact is still understudied [2].

There are common risk factors, such as hospitalisation in the intensive care unit (ICU), chronic respiratory diseases, corticosteroid therapy or intubation and mechanical ventilation [2] that could serve as the field for a co-infection by SARS-CoV-2 and an invasive fungus. As in cases of severe influenza and the known co-morbidity with invasive pulmonary aspergillosis [3,4], cases of patients hospitalised in the ICU for coronavirus disease 2019 (COVID-19) with acute respiratory distress syndrome (ARDS), who suffered a co-infection by *Aspergillus*, have already been reported [5,6,7,8].

*Saccharomyces cerevisiae* has been a well-known and emerging agent of invasive fungal infection since the 1990s in immunocompromised or critically ill patients [9]. While it is a known coloniser or even as part of the normal flora [10] and it is often used in probiotic preparations for the prevention or treatment of various diarrheal disorders [11], it can cause several types of deep infections, most importantly fungaemia [9].

We report two cases of critically ill patients who had to be hospitalised in the ICU due to COVID-19, received *Saccharomyces cerevisiae* supplementation because of diarrhea, and subsequently developed a *Saccharomyces cerevisiae* bloodstream infection. Informed consent was acquired from both patients with opt out possibility.

## 2. Case Reports

They concern two male patients, 76 and 73 years old, with no other underlying diseases apart from regulated arterial hypertension (both of them) and diabetes (the second). The first one was also an ex-smoker.

Both patients presented fever, dyspnea and hypoxia, while the second also had a nonproductive cough. They were admitted for hospitalisation by different departments of internal medicine. In two days’ time, due to the worsening of their clinical condition and the concomitant development of ARDS, they had to be intubated and subsequently transferred to the ICU.

Meanwhile, the molecular testing for SARS-CoV-2 was found positive in both patients’ upper and lower respiratory specimens by the use of real time RT-PCR methods (diagnostic detection protocol for 2019-nCoV, Charité, Berlin, via EVAg and/or VIASURE SARS-CoV-2 detection kit, CerTest Biotec, SL, Zaragoza, Spain). The first patient was found positive again ten and fifteen days later, while he became negative almost twenty-five days later. The second patient was positive when retested on the fifteenth day and negative twenty days later.

During their stay at the ICU they had several successive positive blood cultures for *Staphylococcus hominis* and *Acinetobacter baumannii* first and *Staphylococcus epidermidis* and *Acinetobacter baumannii* second. Moreover, *Acinetobacter baumannii* and *Pseudomonas aeruginosa* were isolated from the cultures of their bronchial secretions.

The first patient, from the day of his admission in the ICU and for ten days thereafter, had a positive blood culture for *Staphylococcus hominis* and, at day 26, for *Acinetobacter baumannii*, which continued even up to day 45. Meanwhile, at day 11, he presented a positive bronchial culture for *Pseudomonas aeruginosa* and, from day 26 and thereafter, for *Acinetobacter baumannii.*

The second patient had a positive blood culture at day 10 by *Acinetobacter baumannii* and at day 15 by *Staphylococcus epidermidis*. The presence of *Acinetobacter baumannii* relapsed at day 18, while all blood cultures became negative at day 30. He also had positive bronchial cultures for *Acinetobacter baumannii* between days 10 and 15 and again from day 18 to day 30.

For all the aforementioned blood and bronchial infections, the two patients received empirical, as well as documented (by culture and sensitivity testing), treatment with several antibiotics, such as piperacillin–tazobactam, moxifloxacin, linezolid, azithromycin, meropenem, colistin, daptomycin and tigecycline. The sequence of the relevant antimicrobials is seen in Table 1. In addition to that, both patients were under treatment with oseltamivir and hydroxychloroquine.

Moreover, the two patients developed a gradual worsening of their renal function and had to undergo several sessions of haemodialysis. The decrease in the kidney capacity could be attributed to both the accumulative nephrotoxicity of several of the antimicrobials, as well as to the COVID-19 itself [12].

Both patients developed diarrhea and were prophylactically treated with Ultra-Levure (preparation of *Saccharomyces cerevisiae* (*boulardii*)) at 250 to 500 mg/day.

The first patient, thirty-five days after his admission at the ICU, while febrile (38–38.5 °C), suffered a bloodstream infection by *Saccharomyces cerevisiae* (Table 1). The same happened with the second patient, fifteen days after his admission to the ICU. Both episodes were possibly related to the use of Ultra-Levure, as they occurred four days after its initiation in the first case and six days in the second one. Initially and before the fungal identification and the sensitivity testing, they were treated with anidulafungin and afterwards with fluconazole. Blood cultures became negative three to four days later, while the treatment with fluconazole continued for fourteen days. Blood cultures were taken daily, from the first positive up to the first negative result, while patients remained fungaemic in the first two days of their antifungal treatment.

After the incubation of the blood cultures, positive direct microscopy and inoculation on Sabouraud dextrose agar with chloramphenicol 0.05% (Conda Pronadisa, Madrid, Spain) and malt extract agar (Sigma-Aldrich Co., St. Louis, MO, USA) at 30 °C and 35 °C, the use of germ tube testing and CHROMagar Candida (Paris, France) and biochemical testing by API ID 32C (bioMérieux SA, Marcy l’ Etoile, France), the two strains were phenotypically identified as *Saccharomyces cerevisiae.*

Further on, both identifications were molecularly confirmed by the amplification and sequencing of the internal transcribed spacer 1 (ITS1) region of the fungal ribosomal DNA (Gen Bank Accession Numbers: MT527544 and MT522376). Both sequences presented a 100% alignment between each other, as well as with the sequence of the strain used in the specific preparation of Ultra-Levure, providing arguments for the genetic relatedness and similarity of all three of them.

A sensitivity testing was attempted by ATB™ Fungus 3 (bioMérieux SA, Marcy l’ Etoile, France) for amphotericin B, flucytosine, fluconazole, itraconazole and voriconazole and MIC (minimum inhibitory concentration) Test Strips (Liofilchem srl, Roseto degli Abruzzi, Italy) for posaconazole and anidulafungin, although there are possible difficulties concerning the growth of *Saccahromyces* and, mainly, *boulardii* on RPMI agar [9]. The growth on RPMI agar was slightly delayed, but after two days of incubation there was a slight, yet adequate, growth that permitted the read.

The MICs for the first isolate were 4 μg/mL for flucytosine, 1.0 μg/mL for amphotericin-B, 0.5 μg/mL for itraconazole, 4.0 μg/mL for fluconazole, 0.125 μg/mL for voriconazole, 0.032 μg/mL for posaconazole and 0.047 μg/mL for anidulafungin, while, for the second one, they were 4 μg/mL for flucytosine, 0.5 μg/mL for amphotericin-B, 0.5 μg/mL for itraconazole, 4.0 μg/mL for fluconazole, 0.125 μg/mL for voriconazole, 0.064 μg/mL for posaconazole and 0.002 μg/mL for anidulafungin. Although there are not defined clinical breakpoints for *Saccharomyces*, the above results indicate a probable in vitro sensitivity to flucytosine, amphotericin-B, fluconazole, voriconazole, posaconazole and anidulafungin.

In both patients, no fungal presence was found in any of the bronchial cultures.

Gradually, both patients showed clinical improvement and were transferred again to the department of internal medicine of the hospital. The first patient, after almost eighty days of hospitalisation, was discharged from the hospital, while the second had to be transferred to a regional teaching hospital due to complications with his tracheostomy.

## 3. The ICU

Both described patients were hospitalised in a small and relatively new intensive care unit with four beds. Due to the outbreak of COVID-19, two more beds, exclusively for these patients, were added. These beds were separated from the rest of the ICU and from one another, while separate nursing staff were provided for each COVID-19 patient. Moreover, all necessary, very strict measures were taken in order to maintain “sterile” conditions between the patients.

According to the experience of the medical staff of the unit and the well-recorded data, no case of fungaemia due to *Saccharomyces* had occurred for at least the last four years in this ICU, despite the fact that it was a long lasting and common practice to use preparations of the fungus for the prophylaxis of patients under antibiotics and concomitant diarrhea. During the last four years, almost eighty patients per year (320 patients in total) were to in this specific ICU and at least half of them were under prophylactic preparations of *Saccharomyces.* This was the first time that such a fungaemia occurred and only during the hospitalisation of these first two COVID patients.

## 4. Discussion

Apart from the devastating consequences of SARS-CoV-2 on the respiratory function, there are also pronounced effects on the absolute numbers of lymphocytes, leading to lymphopenia and an increase in several cytokines and inflammatory markers [13,14,15]. Lymphopenia could be attributed to the virus directly or the white blood cell redistribution, as the T_CD8_ cells have a major role in the clearing of the virus from the pulmonary tissue. However, these cells can be relatively dysfunctional due to the highly produced epithelial cytokines and the impairment of their function could affect the function of dendritic cells and macrophages [16,17,18]. All of the above suggest immune dysfunction and, at least, a host immune imbalance to several extents [19].

The administration of probiotic preparations containing live yeasts, like *Saccharomyces,* may pose a high risk to patients suffering from immune deficiencies due to malignancies or immunosuppressive treatment. Moreover, oral mucositis or ulcers may lead to yeast translocation into the bloodstream of such patients [20,21]. Central venous catheters in critically ill patients could serve as the site of entry due to hand transmission [22,23], although the main portal of entry for invasive infections by *Saccharomyces cerevisiae* is supposed to be digestive [9]. Further on, nosocomial acquisition may occur from patients hospitalised in the same unit [24]. Yeasts persist on room surfaces and at distances of 1 m after the opening of the capsule for administration through the nasogastric tube, while they can also be detected on the hands of healthcare workers [25].

*Saccharomyces boulardii*, which is used in commercial probiotic preparations is considered to be an invalid taxon and either a subtype or a variety of *Saccharomyces cerevisiae* [9,26,27], in fact identical to a particular strain of the latter [28]. Fungaemia by *S. boulardii*, after probiotic use, is more often seen in critically ill patients in the ICU rather than in typical immunodeficient patients [9,20,28]. However, this could be attributed to the prophylactic use of antifungals as routine treatment in immunocompromised, such as oncohaematological, patients [20].

In the described cases, it is interesting that, although the probiotic preparations of *Saccharomyces cerevisiae*/*boulardii* were used for many years as protective agents in this specific ICU and even during the same period of hospitalisation of other patients, only the two specific patients with COVID-19 presented a bloodstream infection. The fact that they were completely separated from one another and the other patients of the ICU and were treated by separate nursing staff, with all the recommended precautions for the avoidance of SARS-CoV-2 transmission, extremely reduces the chances of being contaminated by manipulations or acquisition from other patients or personnel and makes a connection to the probiotic preparations more possible.

In addition, the fact that both fungaemias occurred four to six days after the initiation of Ultra-Levure makes even more possible the connection of the infection to the use of the specific preparation and the concomitant fungal translocation from the gastrointestinal tract to the bloodstream.

Yeast overgrowth and gastrointestinal (GI) leakage, caused by either direct or indirect gastrointestinal injury, could be important pathogenic factors for invasive mycoses. Among other factors, intestinal surgery, haemodialysis, intensive chemotherapy and sepsis could play important roles in the aforementioned GI leakage. Moreover, there are indications that the occurrence of fungal translocation through mucosal barrier damage, as indirectly calculated by the measurement of serum (1→3) β-d-glucan, is correlated to Sepsis-Related Organ Failure Assessment score (SOFA score) and the gravity of the disease in terms of septic shock [29,30].

It is reported that the sequencing of the ITS region of the fungal ribosomal DNA cannot possibly discriminate *S. boulardii* from some *S. cerevisiae* strains [9,31]. However, herein, the results of a 100% alignment between the ITS1 sequences of the patients’ strains and the strain used in the specific preparation of Ultra-Levure, combined with the clinical data, show a good similarity and provide arguments for the genetic relatedness of all three of them.

Although further data and observations are needed, the occurrence of the described cases of two patients suffering from severe COVID-19, with long periods of hospitalisation in the ICU and concomitant bloodstream infection by *Saccharomyces cerevisae,* indicates the need for cautious use of the relevant probiotic preparations in COVID-19 patients.

## Figures and Tables

**Table 1:** Antimicrobials, duration of treatment, Sepsis-Related Organ Failure Assessment scores (SOFA scores), laboratory values and fever on indicative days.

Patient				*Saccharomyces* in Blood Culture		
**Patient 1**	Day 1	Day 11	Day 26	Day 35	Day 45	
	Piperacillin-tazobactam and Linezolid (14 days) Moxifloxacin (10 days) Azithromycin (5 days)	Meropenem (14 days)	Colistin and Tigecycline (21 days)	Anidulafungin (10 days)	Fluconazole (14 days)	
	T: 38.5 °C WBC: 7500/mL Neut.: 92% CRP: 39.5 SOFA: 9			T: 38 °C WBC: 9200/mL Neut.: 50% CRP: 21.6 PCT: 1.04 SOFA: 3	T: 37.5 °C WBC: 9300/mL Neut.: 58% CRP: 8.6 PCT: 0.66 SOFA: 5	
			***Saccharomyces* in Blood Culture**			
**Patient 2**	Day 1	Day 10	Day 15	Day 18	Day 23	Day 30
	Piperacillin-tazobactam (12 days) Linezolid (14 days) Moxifloxacin (10 days) Azithromycin (3 days)	Meropenem (7 days) Colistin (21 days)	Anidulafungin (7 days) Daptomycin (7 days)	Anidulafungin Tigecycline (7 days)	Fluconazole (14 days) Tigecycline Linezolid (8 days)	
	T: 39.0 °C WBC: 8900/mL Neut.: 93% CRP: 26.54 PCT: 0.9 SOFA: 6		T: 38.3 °C WBC: 17500/mL Neut.: 82% CRP: 13.15 PCT: 1.15 SOFA: 9		SOFA: 9	T: 37.0 °C WBC: 7600/mL Neut.: 72% CRP: 6.0 PCT: 0.9 SOFA: 4

Temperature (T), white blood cell count (WBC), neutrophils (Neut.), C-reactive protein (CRP, 0–0.5 mg/dL), procalcitonin (PCT, 0–0.5 ng/mL), Sepsis-Related Organ Failure Assessment score (SOFA score).
